# Relationships among perceived stress, anxiety, and well-being in Chinese hospital staff: the mediating role of self-efficacy and the moderating role of empathy

**DOI:** 10.3389/fpsyg.2026.1740682

**Published:** 2026-02-11

**Authors:** Mingji Li, Guohai Yang, Xianhua Xu, Insoo Oh, Shijie Xu

**Affiliations:** 1 Department of Education, College of Education, Ewha Womans University, Seoul, Republic of Korea; 2 Department of Sociology, Hong Kong Shue Yan University, Hong Kong, Hong Kong SAR, China; 3 Department of Pathology, Affiliated Cancer Hospital of Hainan Medical University, Haikou, Hainan, China; 4 Institute of Human Behavioral Medicine, Medical Research Center, Seoul National University, Seoul, Republic of Korea

**Keywords:** perceived stress, anxiety, self-efficacy, empathy, well-being, Chinese hospital staff

## Abstract

**Objective:**

This study aimed to investigate whether self-efficacy mediates the relationship between perceived stress/anxiety and well-being and whether empathy moderates the association between self-efficacy and well-being.

**Methods:**

A cross-sectional study was conducted among 543 full-time hospital staff members at Hainan Cancer Hospital in China. Participants completed the Perceived Stress Scale (PSS-10), the Generalized Anxiety Disorder Scale (GAD-7), the General Self-Efficacy Scale (GSES), the Interpersonal Reactivity Index (IRI), and the WHO-5 Well-being Index. A moderated mediation analysis was performed using the SPSS PROCESS Macro.

**Results:**

Mediation analysis indicated that self-efficacy partially mediated the relationship between perceived stress/anxiety and well-being. Empathy moderated the relationship between self-efficacy and well-being, enhancing its protective effects. Specifically, individuals with higher levels of empathy exhibited a stronger positive association between self-efficacy and well-being, highlighting the synergistic role of empathy in mitigating the adverse effects of stress and anxiety.

**Conclusion:**

These findings underscore the importance of interventions targeting self-efficacy and empathy to improve well-being among hospital staff. This study contributes to the growing body of literature on occupational mental health in high-stress healthcare settings.

## 1 Introduction

Well-being critically influences healthcare professionals’ job satisfaction, performance, and the quality of care they provide to patients (Almeida et al., 2024). Low levels of well-being among hospital staff are associated with poorer patient safety, higher rates of medical errors, and weaker teamwork and communication (Hall et al., 2016; Lee et al., 2013). Studies in China have identified several key factors influencing hospital staff well-being, including social support (Lu et al., 2023), occupational burnout (Xiao et al., 2022), and exposure to workplace violence (Tang and Thomson, 2019). Chinese hospital staff face distinctive occupational challenges. Chinese healthcare workers report lower social support, weaker workplace belonging, poorer role clarity, and greater job insecurity compared with their counterparts in Canada, Spain, France, Germany, Sweden, and Turkey (Wang F. et al., 2025). Additionally, nurses from Eastern cultural backgrounds exhibited higher burnout rates than their Western counterparts during the COVID-19 pandemic (Frey et al., 2025). These contextual factors underscore the unique occupational pressures on Chinese hospital staff and provide a rationale for the present study’s focus on this population. Understanding these dynamics is essential for developing effective interventions to sustain healthcare system performance.

Hospital staff frequently experience stress and anxiety due to heavy workloads, doctor-patient relationships, and workplace pressures (Liu et al., 2024; Liu et al., 2020; Zhang et al., 2024), significantly impacting their overall well-being. Previous studies have reported that stress and anxiety are closely associated with well-being. For example, psychological stress and anxiety substantially affect mental health and overall well-being in high school and university students (Freire et al., 2016; Moeini et al., 2008). Similarly, dental students’ well-being is influenced by anxiety and stress (Stormon et al., 2022). Notably, the incidence of depression and anxiety was very high among healthcare workers during the COVID-19 pandemic, which significantly reduced their psychological well-being (Dhingra and Dhingra, 2020; Xu et al., 2020). These findings suggest that individuals in high-pressure environments are more likely to experience diminished well-being consistent with cognitive appraisal theory, which posits that individuals’ subjective evaluation of situations determines their emotional responses (Lazarus and Folkman, 1984). However, the psychological mechanisms that mediate or moderate these relationships, particularly in high-pressure healthcare environments such as Chinese hospitals, remain insufficiently understood.

Self-efficacy, defined as an individual’s belief in their capacity to achieve goals through their actions (Bandura et al., 1997), may play a key role in mitigating the effects of stress and anxiety on the well-being of hospital staff. By strengthening an individual’s sense of control in difficult circumstances, self-efficacy helps manage emotional reactions and encourages adaptive coping strategies (Hobfoll, 1989). Self-efficacy, in turn, enhances individuals’ perceived control over stressors, thereby fostering better psychological outcomes (Schwarzer and Hallum, 2008). For instance, a study of 240 female healthcare workers found that higher anxiety levels corresponded to lower self-efficacy, suggesting that interventions to bolster self-efficacy may benefit those with high anxiety (Muñoz et al., 2018). Another study demonstrated that self-efficacy fully mediated the relationship between job stress and psychological well-being among home-visiting nursing care workers during the COVID-19 pandemic (Kim et al., 2022). Based on these findings, we hypothesized that high stress and anxiety may reduce self-efficacy, thereby decreasing well-being.

The mediation effect of self-efficacy on well-being may be moderated by psychological factors, with empathy being particularly significant. Empathy is defined as the ability to understand and vicariously experience others’ emotional states (Davis, 1983). Higher empathy is associated with increased psychological well-being among emergency nurses, medical residents, and surgical residents (Bourgault et al., 2015; Graziano et al., 2024; Swenson et al., 2024; Vinh-Long et al., 2019). However, while high empathic ability can enhance well-being by promoting emotional connections and professional fulfillment, excessive empathy may contribute to compassion fatigue and emotional exhaustion (Cadet and Sainfort, 2023; Wang R. et al., 2025). Furthermore, for individuals with high self-efficacy, greater empathy may enhance well-being by strengthening patient relationships and job satisfaction, whereas low empathy may reduce these benefits by limiting emotional resilience in interpersonal interactions. Conversely, when self-efficacy is low, high empathy may increase emotional exhaustion due to heightened sensitivity to patient distress, while low empathy may hinder well-being due to difficulties in social engagement. Thus, we hypothesize that empathy, as a psychological resource, moderates the relationship between self-efficacy and well-being.

**Figure 1:**
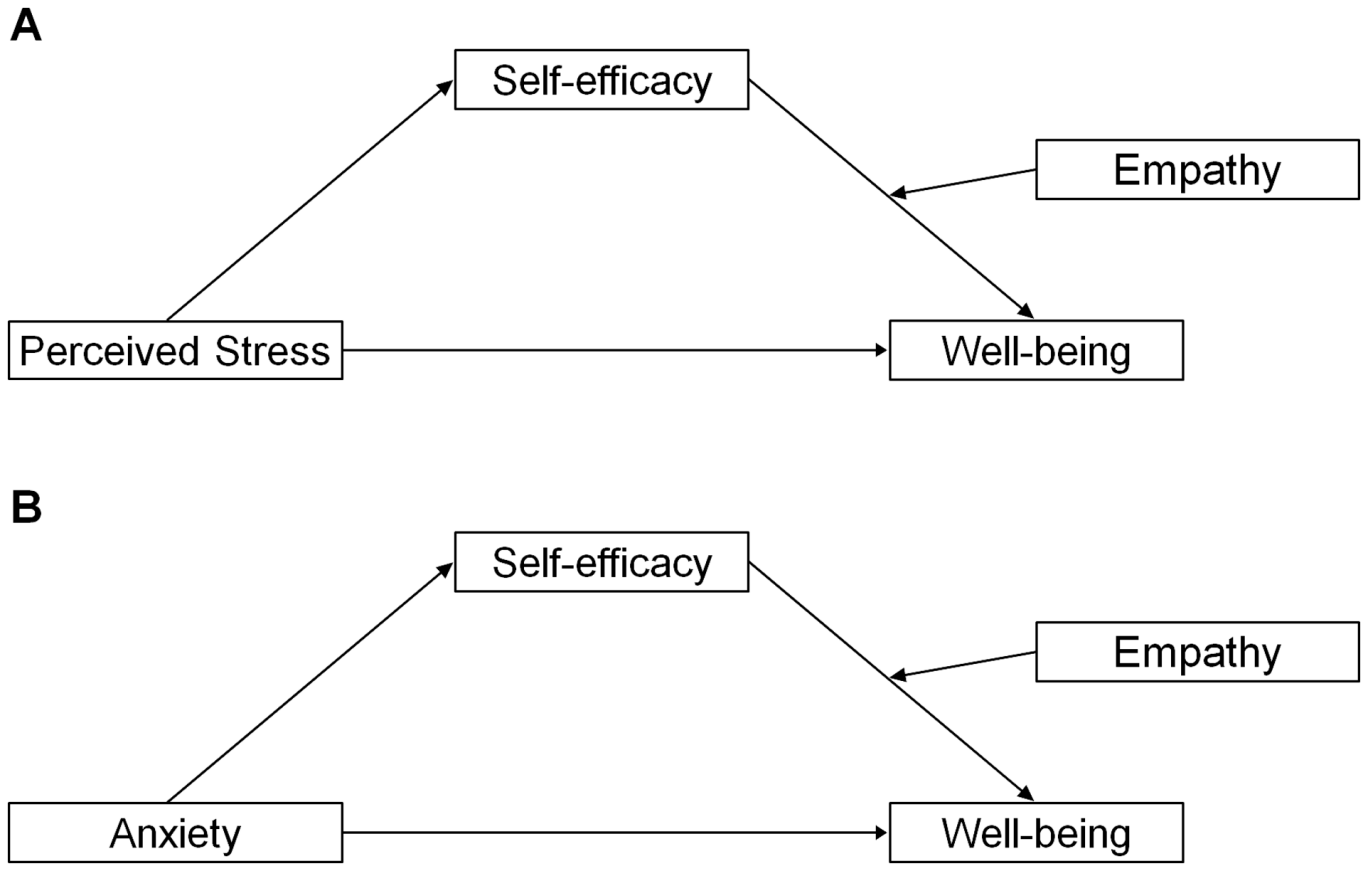
The proposed moderated mediation models of the relationships among perceived stress **(A)**, anxiety **(B)**, self-efficacy (mediator), empathy (moderator), and well-being.

Based on the theoretical framework and empirical evidence discussed above, this study aimed to explore the relationships among perceived stress, anxiety, self-efficacy, empathy, and well-being among Chinese hospital staff. Specifically, it investigates whether self-efficacy mediates the effects of perceived stress and anxiety on well-being, and whether empathy moderates this mediation pathway. This study contributes to the literature by elucidating the conditional mechanisms through which psychological resources buffer occupational stress among Chinese hospital staff, offering theoretical and practical value for developing targeted interventions. Although prior research has established the adverse effects of perceived stress on well-being, few studies have examined this relationship within a mediated moderation framework in which self-efficacy functions as a mediating mechanism while empathy simultaneously operates as a contextual moderator. Thus, this approach represents a distinctive contribution of the present study. The need for such investigation is further underscored by evidence that Chinese hospital staff experience comparatively high occupational stress alongside relatively low well-being. In work environments where stress reduction is often challenging, identifying psychological mediators and moderators that attenuate its negative impact on well-being helps inform the development of effective intervention strategies to promote the well-being of healthcare personnel. The hypothesized moderated mediation model is presented in Figure 1. The following three hypotheses were proposed:

*H1*: Perceived stress and anxiety negatively affect well-being among healthcare professionals.

*H2*: Self-efficacy mediates the relationship between perceived stress/anxiety and well-being.

*H3*: Empathy moderates the indirect effect of self-efficacy on well-being, strengthening the positive relationship between self-efficacy and well-being.

## 2 Materials and methods

### 2.1 Participants and procedure

This study employed a cross-sectional research design. A total of 600 full-time hospital staff were recruited from a tertiary hospital in Hainan province, China. The hospital is a comprehensive cancer-focused tertiary institution in which staff frequently encounter patients and families experiencing high levels of emotional distress and uncertainty, potentially contributing to elevated occupational stress. The hospital also includes non-oncology departments, and the study sample, therefore, comprised staff from oncology and non-oncology units. Participants were recruited through convenience sampling. An internal hospital email introducing the study was distributed to all staff. Eligibility was restricted to full-time employees who voluntarily expressed interest in participating and provided informed consent. Part-time or temporary employees and those who declined to participate were excluded. Consenting participants subsequently received an online survey link, and a paper-based version of the questionnaire was also made available upon request. Participation was entirely voluntary and anonymous, and all personal information was de-identified prior to analysis. Data collection occurred between March and May 2025 using a structured self-report questionnaire. Questionnaires were excluded from analysis if they contained more than 10% missing data, exhibited uniform response patterns across all items (indicative of inattentive responding), or were otherwise incomplete. After removing invalid questionnaires, 543 valid responses were retained, yielding a valid response rate of 90.5%.

Participant demographics were as follows: 168 men (30.94%) and 375 women (69.06%); 59 participants were under 25 years (10.87%), 371 were 25–35 years (68.32%), 86 were 36–45 years (15.84%), and 27 were over 45 years (4.97%). Marital status included 210 unmarried individuals (38.67%), 293 married individuals (53.96%), and 40 divorced or widowed individuals (7.37%). Occupationally, participants comprised 170 doctors (31.31%), 249 nurses (45.86%), and 124 non-medical staff (22.84%). Participants comprised 64 high school graduates (11.79%), 394 bachelor’s degree holders (72.56%), 58 master’s degree holders (10.68%), and 27 doctoral degree holders (4.97%). Regarding work experience, 47 had worked less than 2 years (8.66%), 194 for 2–5 years (35.73%), 174 for 6–10 years (32.04%), 80 for 11–15 years (14.73%), and 48 had worked more than 15 years (8.84%). Weekly working hours were < 40 h for 68 participants (12.52%), 40–48 h for 339 participants (62.43%), and > 48 h for 136 participants (25.05%). Monthly income per capita was < 5,000 CNY for 158 participants (29.10%), 5,000–10,000 CNY for 292 participants (53.78%), 10,000–20,000 CNY for 75 participants (13.81%), and > 20,000 CNY for 18 participants (3.31%). Details are presented in Table 1.

**Table 1:** Descriptive characteristics of participants (*n* = 543).

Variable	Category	Frequency (*n*)	Percentage (%)
Gender	Men	168	30.94%
	Women	375	69.06%
Age (years)	< 25	59	10.87%
	25–35	371	68.32%
	36–45	86	15.84%
	> 45	27	4.97%
Marital status	Unmarried	210	38.67%
	Married	293	53.96%
	Divorced or widowed	40	7.37%
Occupation	Doctor	170	31.31%
	Nurse	249	45.86%
	Non-medical staff	124	22.84%
Educational level	High school	64	11.79%
	Bachelor’s degree	394	72.56%
	Master’s degree	58	10.68%
	Doctor’s degree	27	4.97%
Years of work experience	< 2	47	8.66%
	2–5	194	35.73%
	6–10	174	32.04%
	11–15	80	14.73%
	> 15	48	8.84%
Weekly working hours	< 40	68	12.52%
	40–48	339	62.43%
	> 48	136	25.05%
Income	< 5,000	158	29.10%
	5,000 − 10,000	292	53.78%
	10,000 − 20,000	75	13.81%
	> 20,000	18	3.31%

### 2.2 Measures

#### 2.2.1 Perceived stress scale (PSS-10)

The PSS-10 is a widely used instrument for assessing perceived stress (Cohen et al., 1983). The scale comprises 10 items rated on a Likert scale from 0 (Never) to 4 (Very Often), including four positively worded and six negatively worded items. Total scores ranged from 0 to 40, with higher scores indicating higher perceived stress. The Chinese version of the PSS-10 was employed in this study. Previous studies have demonstrated satisfactory validity among Chinese nurses (Cronbach’s *α* = 0.86; Du et al., 2023) and university students (Cronbach’s α = 0.85; Lu et al., 2017). The Chinese version of the PSS-10 demonstrated satisfactory internal consistency in this study (Cronbach’s *α* = 0.853).

#### 2.2.2 Generalized anxiety disorder (GAD-7)

The GAD-7 was used to assess the severity of anxiety symptoms during the past 2 weeks (Spitzer et al., 2006). The questionnaire consists of seven items rated on a 4-point Likert scale (0–3). The Chinese version of the GAD-7 has demonstrated satisfactory psychometric properties among Chinese medical university students (Cronbach’s *α* = 0.93; Zhang et al., 2021) and demonstrated good internal consistency in the present sample (Cronbach’s *α* = 0.923).

#### 2.2.3 General self-efficacy scale (GSES)

The GSES was used to measure general self-efficacy, reflecting participants’ confidence in handling difficulties or frustration (Zhang and Schwarzer, 1995). The scale consists of 10 items rated on a 4-point Likert scale (1–4), with higher scores indicating higher self-efficacy. The Chinese version of the GSES has demonstrated reliability, validity, and stable factor structure among Chinese students (Cronbach’s *α* = 0.91; Zeng et al., 2022). In the present study, the GSES demonstrated excellent internal consistency (Cronbach’s *α* = 0.935).

#### 2.2.4 Empathy (interpersonal reactivity index-C)

The IRI evaluates empathy (Davis, 1980) using 22 items, rated on a 5-point Likert scale (0–4), with total scores ranging from 0 to 88; higher scores indicate higher empathy. The scale is divided into four sub-dimensions: perspective-taking, personal distress, fantasy, and empathic concern. The Chinese version of the IRI has demonstrated satisfactory reliability and construct validity among Chinese people (Cronbach’s *α* = 0.750; Zhang et al., 2010). In the present study, internal consistency was excellent (Cronbach’s α = 0.930).

#### 2.2.5 Well-being (World Health Organization, WHO-5)

The WHO-5 Well-Being Index, originally developed by the World Health Organization (1998), was used to assess participants’ psychological well-being over the past 2 weeks. The scale consists of 5 items rated on a 6-point Likert scale (0–5), with total scores ranging from 0 to 25; higher scores indicate greater well-being. The Chinese version of the WHO-5 has demonstrated satisfactory reliability and validity among Chinese populations (Cronbach’s α = 0.81–0.85; Fung et al., 2022). In this study, the WHO-5 demonstrated excellent internal consistency (Cronbach’s α = 0.954).

### 2.3 Statistical analysis

Data analysis was performed using SPSS version 22.0 (IBM Corp., Armonk, NY, USA) and PROCESS Macro for SPSS 4.3 (Hayes, 2017). The possibility of common method bias was assessed using Harman’s single-factor test, with a threshold of less than 40% for the total variance explained by the first factor. Pearson correlation analysis was calculated to examine the relationships among perceived stress, anxiety, self-efficacy, empathy, and well-being. Mediation analysis was conducted using Model 4 of the PROCESS Macro, with perceived stress and anxiety as predictors, self-efficacy as the mediator, and well-being as the outcome variable. A moderated mediation model was subsequently analyzed using Model 14 of the PROCESS Macro to assess whether empathy moderated this mediation process. To further explore the moderating effect, a simple slope analysis was conducted to evaluate the relationship between self-efficacy and well-being at three levels of empathy: low (1 SD below the mean), mean, and high (1 SD above the mean). The corresponding regression lines were plotted. Control variables, including gender, age, marital status, occupation, educational level, years of work experience, weekly working hours, and income, were included as covariates in the models. Bootstrapping with 5,000 samples and 95% confidence intervals (CIs) was used to estimate standard errors (SEs) of conditional direct and indirect effects. All variables were mean-centered prior to creating interaction terms to reduce multicollinearity.

## 3 Results

### 3.1 Test of common method bias

The results of the Harman’s single-factor test indicated that nine factors had eigenvalues greater than 1. The first factor accounted for 26.623% of the total variance, which is below the critical threshold of 40%, suggesting that common method bias was not a significant concern in this study.

### 3.2 Correlation analysis

The means, standard deviations, and Pearson correlations for the study variables are presented in Table 2. Perceived stress was positively correlated with anxiety (*r* = 0.655, *p* < 0.001) and negatively associated with self-efficacy (*r* = −0.497, *p* < 0.001), empathy (*r* = −0.162, *p* < 0.001), and well-being (*r* = −0.481, *p* < 0.001). Anxiety was also negatively correlated with self-efficacy (*r* = −0.599, *p* < 0.001), empathy (*r* = −0.170, *p* < 0.001), and well-being (*r* = −0.528, *p* < 0.001). Self-efficacy was positively correlated with empathy (*r* = 0.213, *p* < 0.001) and well-being (*r* = 0.515, *p* < 0.001). Additionally, empathy and well-being were positively correlated (*r* = 0.254, *p* < 0.001).

**Table 2:** The correlations between the main study variables.

Variable	*M*	SD	Perceived stress	Anxiety	Self-efficacy	Empathy
Perceived stress	17.93	5.012	–			
Anxiety	7.61	5.896	0.655***	–		
Self-efficacy	22.36	7.431	−0.497***	−0.599***	–	
Empathy	42.23	9.781	−0.162***	−0.170***	0.213***	–
Well-being	15.04	7.104	−0.481***	−0.528***	0.515***	0.254***

*M*, Mean; SD, Standard Deviation; ***p < 0.001.

### 3.3 Mediation effect of self-efficacy

Mediation analysis was conducted using model 4 of the PROCESS Macro in SPSS, with perceived stress and anxiety as independent variables, well-being as the dependent variable, and self-efficacy as the mediator (Table 3). Gender, age, marital status, occupation, educational level, years of work experience, weekly working hours, and income were included as covariates. After controlling for gender and demographic variables, perceived stress significantly negatively predicted well-being (Model 1: *β* = −0.479, *t* = −12.593, *p* < 0.001) and self-efficacy (Model 2: *β* = −0.488, *t* = −13.093, *p* < 0.001). When self-efficacy was included as a mediator, perceived stress continued to significantly negatively predict well-being (Model 3: *β* = −0.296, *t* = −7.268, *p* < 0.001), while self-efficacy significantly positively predicted well-being (Model 3: *β* = 0.375, *t* = 9.112, *p* < 0.001).

**Table 3:** The mediation effect of perceived stress and anxiety on well-being through self-efficacy.

Variables	Model 1 (Well-being)		Model 2 (Self-efficacy)		Model 3 (Well-being)	
	*β*	*t*	*β*	*t*	*β*	*t*
Perceived stress → self-efficacy → well-being						
Perceived stress	−0.479	−12.593***	−0.488	−13.093***	−0.296	−7.268***
Self-efficacy	–	–	–	–	0.375	9.122***
*R^2^*	0.247	–	0.275	–	0.348	–
*F*	19.381***	–	22.410***	–	28.454***	–
Anxiety → self-efficacy → well-being						
Anxiety	−0.527	−14.275***	−0.595	−17.267***	−0.338	−7.667***
Self-efficacy	–	–	–	–	0.318	7.166***
*R^2^*	0.293	–	0.385	–	0.355	–
*F*	24.518***	–	37.092***	–	29.287***	–

*β*, Standardized coefficient; *R*^2^, Coefficient of Determination; ***p < 0.001.

For anxiety, with gender and demographic variables controlled, it significantly negatively predicted well-being (Model 1: *β* = −0.527, *t* = −14.275, *p* < 0.001) and self-efficacy (Model 2: *β* = −0.595, *t* = −17.267, *p* < 0.001). Self-efficacy positively predicted well-being (Model 3: *β* = 0.318, *t* = 7.166, *p* < 0.001), and the direct effect of anxiety on well-being remained significant (Model 3: *β* = −0.338, *t* = −7.667, *p* < 0.001).

The significance of the mediating effects was further tested using bootstrapping analysis (Table 4). For perceived stress, the total effect, direct effect, and indirect effects on well-being via self-efficacy were −0.679 [95% CI (−0.784, −0.573)], −0.419 [95% CI (−0.532, −0.306)], and −0.259 [95% CI (−0.333, −0.193)], respectively. For anxiety, the corresponding effects were −0.635 [95% CI (−0.723, −0.548)], −0.407 [95% CI (−0.512, −0.303)], and −0.228 [95% CI (−0.300, −0.160)], respectively. These results indicate that self-efficacy partially mediates the relationship between perceived stress/anxiety and well-being.

**Table 4:** Mediation model effects.

Variable relationship	Effect	*SE*	*t*	95% CI	
Perceived stress → self-efficacy → well-being					
Total effect	−0.679	0.054	−12.593***	−0.784	−0.573
Direct effect	−0.419	0.058	−7.268***	−0.532	−0.306
Indirect effect	−0.259	0.036	-	−0.333	−0.193
Anxiety → self-efficacy → well-being					
Total effect	−0.635	0.045	−14.275***	−0.723	−0.548
Direct effect	−0.407	0.053	−7.667***	−0.512	−0.303
Indirect effect	−0.228	0.036	-	−0.300	−0.160

SE, Standard Error; CI, Confidence Interval; ***p < 0.001.

### 3.4 Moderated mediation effect of empathy

Moderated mediation analysis was conducted using Model 14 of the PROCESS Macro in SPSS. Empathy was entered as a moderator in the association between self-efficacy and well-being (Table 5). After controlling for gender and demographics, perceived stress was a significant predictor of self-efficacy (Model 1: *B* = −0.724, *t* = −13.093, *p* < 0.001). In addition, perceived stress (Model 2: *B* = −0.416, *t* = −7.362, *p* < 0.001) and self-efficacy (*B* = 0.326, *t* = 8.345, *p* < 0.001) significantly predicted well-being. The interaction between self-efficacy and empathy also significantly predicted well-being (*B* = 0.011, *t* = 3.452, *p* < 0.01).

**Table 5:** The moderated mediation model with self-efficacy as a mediator and empathy as a moderator.

Variables	Model 1 (Self-efficacy)		Model 2 (Well-being)	
	*B*	*t*	*B*	*t*
Perceived stress → self-efficacy → well-being				
Perceived stress	−0.724	−13.093***	−0.416	−7.362***
Self-efficacy	–	–	0.326	8.345***
Empathy	–	–	0.093	3.634***
Self-efficacy × empathy	–	–	0.011	3.452**
*R^2^*	0.275	–	0.379	–
*F*	22.410***	–	26.905***	–
Anxiety → self-efficacy → well-being				
Anxiety	−0.750	−17.267***	−0.393	−7.536***
Self-efficacy	–	–	0.278	6.592***
Empathy	–	–	0.096	3.750***
Self-efficacy × empathy	–	–	0.009	2.840**
*R^2^*	0.385	–	0.381	–
*F*	37.092***	–	27.220***	–

*B*, Unstandardized coefficient; *R*^2^, Coefficient of Determination; **p < 0.01, ***p < 0.001.

The results for anxiety as the independent variable were similar to those observed for perceived stress. With gender and demographic variables controlled, anxiety significantly negatively predicted self-efficacy (Model 1: *B* = −0.750, *t* = −17.267, *p* < 0.001). Moreover, anxiety (Model 2: *B* = −0.393, *t* = −7.536, *p* < 0.001) and self-efficacy (*B* = 0.278, *t* = 6.592, *p* < 0.001) significantly predicted well-being. The interaction between self-efficacy and empathy also significantly influenced well-being (*B* = 0.009, *t* = 2.840, *p* < 0.01). These results indicate that empathy moderates the mediation of self-efficacy in the association between perceived stress/anxiety and well-being.

**Figure 2:**
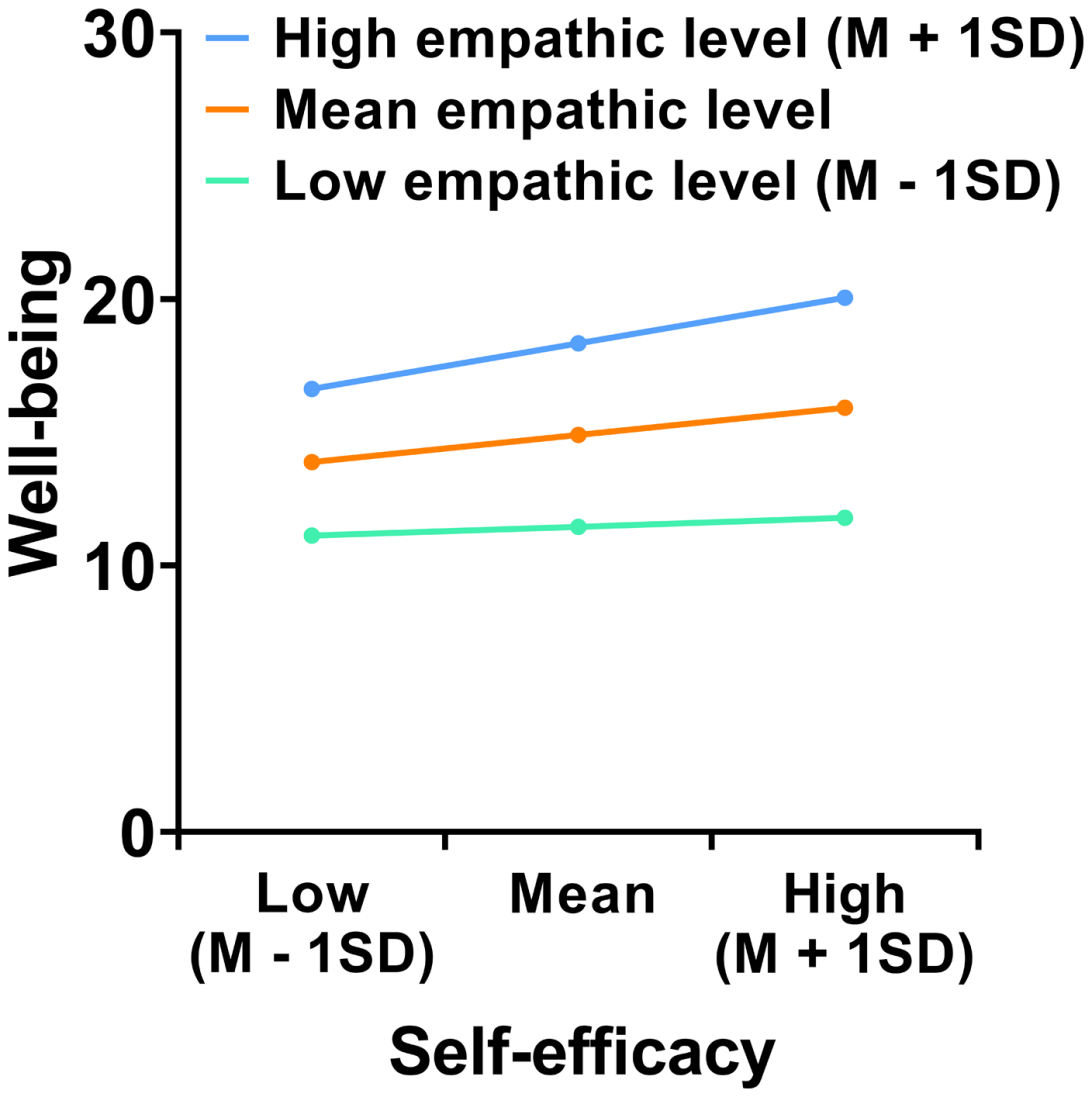
Simple slope analysis illustrating that greater empathy strengthens the positive association between self-efficacy and well-being among hospital staff.

To further examine the moderating effect of empathy levels on the relationship between self-efficacy and well-being, a simple slope analysis was performed (Figure 2), revealing that the relationship between self-efficacy and well-being was stronger among individuals with high levels of empathy (*B* = 0.556, *t* = 11.944, *p* < 0.001) than in individuals with low levels of empathy (*B* = 0.371, *t* = 7.595, *p* < 0.001). Moreover, the conditional indirect effects of perceived stress and anxiety on well-being, mediated by self-efficacy, were also moderated by empathy levels (Table 6). Specifically, the conditional indirect effect of perceived stress on well-being through self-efficacy was significant across all empathy levels [−1 SD, *B* = −0.161, 95% CI (−0.239, −0.090); mean, *B* = −0.236, 95% CI (−0.306, −0.172); +1 SD, *B* = −0.311, 95% CI (−0.400, −0.231)]. As shown in Figure 2, the slopes for the high-empathy group (≥ 1 SD above the mean) and the low-empathy group (≤ 1 SD below the mean) differed significantly. In other words, the effect of self-efficacy on well-being was stronger among individuals with higher empathy than among those with lower empathy. These findings indicate that empathy significantly moderates the relationship between self-efficacy and well-being. The moderated mediation index was −0.008 (95% CI -0.012 to −0.003), which does not include zero, confirming a significant moderated mediation effect. Similarly, the conditional indirect effect of anxiety on well-being via self-efficacy was significant across all levels of empathy [−1 SD, *B* = −0.145, 95% CI (−0.224, −0.066); Mean, *B* = −0.208, 95% CI (−0.281, −0.140); +1 SD, *B* = −0.272, 95% CI (−0.357, −0.186)]. The index of moderated mediation was −0.006 (95% CI -0.011 to −0.002), supporting the presence of moderated mediation. These results indicate that the positive predictive effect of self-efficacy on well-being increased progressively with higher levels of empathy, highlighting empathy’s moderating role in the relationship between self-efficacy and well-being.

**Table 6:** Conditional indirect effect results.

Variables		Effect	Boot SE	95% CI	
Perceived stress → self-efficacy → well-being					
Empathy	−1 SD	−0.161	0.038	−0.239	−0.090
	Mean	−0.236	0.035	−0.306	−0.172
	+1 SD	−0.311	0.043	−0.400	−0.231
Index of moderated mediation		−0.008	0.002	−0.012	−0.003
Anxiety → self-efficacy → well-being					
Empathy	−1 SD	−0.145	0.041	−0.224	−0.066
	Mean	−0.208	0.036	−0.281	−0.140
	+1 SD	−0.272	0.044	−0.357	−0.186
Index of moderated mediation		−0.006	0.002	−0.011	−0.002

SD, Standard Deviation; SE, Standard Error; CI, Confidence Interval.

## 4 Discussion

This study highlights the intricate relationships between perceived stress, anxiety, self-efficacy, empathy, and well-being among hospital staff in China, offering valuable insights into the psychological mechanisms underlying occupational stress and anxiety in high-pressure healthcare environments. The results reveal negative correlations between perceived stress and well-being, as well as between anxiety and well-being. Moreover, self-efficacy mediated the associations between perceived stress/anxiety and well-being, while empathy moderated the relationship between self-efficacy and well-being, confirming our initial hypotheses. Overall, these findings contribute to a deeper understanding of how perceived stress and anxiety influence well-being in healthcare settings.

Consistent with prior research (Liu and Aungsuroch, 2019; Strizhitskaya et al., 2019), this study demonstrates that perceived stress and anxiety significantly predict reduced well-being. Stress and anxiety are inevitable aspects of work environments, particularly in high-pressure occupations such as healthcare, where demands often exceed coping capacities. These findings align with cognitive appraisal theory, which posits that stress arises when individuals perceive environmental demands as exceeding their coping abilities (Lazarus and Folkman, 1984). Chinese nurses are particularly prone to moderate-to-severe stress and anxiety, with reported anxiety rates ranging from 26.4 to 43.4% (Gao et al., 2012; Liu et al., 2024). Mid- to late-career nurses also exhibit higher anxiety sensitivity than early-career nurses (Li et al., 2016). Similarly, Goh et al. (2015) identified ten stressors negatively affecting healthcare workers’ mental health in the United States, including job insecurity (which increases the likelihood of poor health outcomes by approximately 50%) and excessive work hours (linked to an increase of approximately 20% in mortality risk). Furthermore, perceived stress has been shown to be negatively correlated with clinical performance among Chinese nursing trainees (Ye et al., 2018). Therefore, effectively managing perceived stress and anxiety in high-pressure healthcare environments is critical not only for improving the well-being of hospital staff but also for maintaining optimal clinical performance.

Perceived stress and anxiety not only directly reduce well-being but also indirectly reduce it by lowering self-efficacy, thereby mitigating the sense of well-being. Research employing the GSES highlights that healthcare workers with high self-efficacy demonstrate greater problem-solving abilities and emotional regulation when managing occupational stress (Schwarzer and Jerusalem, 1995). These adaptive responses are influenced by workplace factors, including workload intensity, systemic stressors, compensation equity, and collegial support (Hu et al., 2018; Molero et al., 2018). For example, enhancing self-efficacy among healthcare professionals can increase work engagement and personal satisfaction (Cabrera-Aguilar et al., 2023) and mediates the relationship between job stress and burnout, as well as subsequent mental health challenges such as depression and anxiety (Lin et al., 2016). Consistent with these findings, our results show that hospital staff experiencing high stress or anxiety report lower self-efficacy, which reduces well-being. Therefore, interventions that enhance self-efficacy are crucial for improving overall well-being among hospital staff.

The results further demonstrate the moderating role of empathy in the relationship between self-efficacy and well-being. Empathy, as a moderator, indicates that individual differences in empathetic capacity influence the effectiveness of stress-management strategies, suggesting the potential benefits of interventions to strengthen empathy. Moreover, empathy can prevent burnout, enhance the quality of care, and improve job satisfaction (Bogiatzaki et al., 2019; Yu et al., 2022). Healthcare workers with higher empathy may experience greater professional fulfillment, reduced stress, and improved well-being (Decety, 2020). In addition, individuals with high empathy tend to exhibit compassion satisfaction, transpersonal thinking, and altruistic behavior, whereas those with low empathy are more likely to feel compassion fatigue and personal distress (Gleichgerrcht and Decety, 2013). This dynamic aligns with Davis’s (1983) empathy framework, in which cognitive and affective components interact with professional confidence to sustain well-being. Clinicians who strike a balance between empathy and self-efficacy often find fulfillment in patient care, and empathetic interactions are associated with improved patient outcomes (Gerdes and Segal, 2011). However, high-pressure healthcare environments and systemic constraints can disrupt this balance. For example, medical students and residents frequently experience declines in empathy during training (Neumann et al., 2011). Furthermore, interventions such as mindfulness-based stress reduction can alleviate anxiety, enhance self-efficacy, and promote empathic capacity among hospital staff (Shapiro et al., 2005). Promoting empathic competencies may, therefore, improve well-being and performance among healthcare workers.

This study identifies self-efficacy as a mediating variable and empathy as a moderating variable in mitigating the adverse effects of perceived stress and anxiety on well-being among hospital staff, providing actionable insights for designing targeted interventions to support healthcare workers. For example, interventions focused on self-efficacy could include stress management training, goal-setting strategies, and fostering transformational leadership in healthcare settings to enhance mental health and job satisfaction (Nielsen et al., 2009). In addition, empathy can be developed through mindfulness-based intervention, which not only enhances empathetic capacity but also significantly reduces stress and burnout among healthcare workers (Raab, 2014). Similarly, emotional intelligence training can help healthcare workers maintain empathy during high-stress situations (Sharp et al., 2020). Promoting self-regulation skills among healthcare workers further contributes to a better understanding of the factors influencing well-being and the underlying mechanisms, thereby improving strategies for staff support and organizational intervention.

This study has some limitations. First, the cross-sectional nature of this study precludes causal inference. Because temporal ordering among the variables cannot be determined, inverse or bidirectional associations remain plausible. For example, lower well-being may increase perceived stress and anxiety, rather than merely resulting from them. Future longitudinal and prospective studies are needed to clarify causal directions and to examine how self-efficacy and empathy dynamically interact with stressors over time. Second, single-site sampling from Hainan Province limits the generalizability of the findings. Future research should incorporate multi-regional comparisons to account for disparities in healthcare resources and workloads across urban and rural China. Third, although prior studies have conceptualized empathy as a “double-edged sword”—with the potential to contribute to compassion fatigue under specific conditions (Cadet and Sainfort, 2023; Wang R. et al., 2025)—our findings primarily support its protective role. Future research is warranted to identify the circumstances under which empathy may shift from a protective factor to a risk factor. Fourth, the empathy measure used in this study consists of four subscales, which are sometimes grouped into two higher-order dimensions—cognitive empathy (e.g., perspective-taking) and affective empathy (e.g., empathic concern). However, the present study treated empathy as a single composite construct for analytic purposes. Future research should examine the differential effects of these subdimensions to more precisely identify the pathways through which empathy relates to well-being. Fifth, well-being among healthcare workers is influenced by a wide range of individual, interpersonal, and organizational factors that were not fully captured in the present study. Future research should include additional predictors—such as social support, organizational culture, resilience, and coping strategies—which may further explain variance in well-being. Finally, the broad categorization of “hospital staff” overlooks potential differences among doctors, nurses, and non-medical staff, which may influence the relationships between variable. Future studies should recruit larger samples and stratify analyses by occupational subgroups.

## 5 Conclusion

This study demonstrates that perceived stress and anxiety significantly undermine the well-being of Chinese hospital staff, with self-efficacy acting as a partial mediator and empathy serving as a moderator in this relationship. Specifically, self-efficacy mitigates the negative psychological impacts of stress by enhancing an individual’s perceived control over the stressor, whereas empathy, when combined with strong self-efficacy, amplifies this protective mechanism. Based on these findings, hospital staff should be supported through interventions that simultaneously enhance self-efficacy and foster empathy, ultimately promoting their well-being. This approach can strengthen individuals’ ability to cope with mental health challenges and life adversity.

## Data Availability

The original contributions presented in the study are included in the article/Supplementary material, further inquiries can be directed to the corresponding author.
